# Characterisation of the Native Lipid Moiety of *Echinococcus granulosus* Antigen B

**DOI:** 10.1371/journal.pntd.0001642

**Published:** 2012-05-15

**Authors:** Gonzalo Obal, Ana Lía Ramos, Valeria Silva, Analía Lima, Carlos Batthyany, María Inés Bessio, Fernando Ferreira, Gustavo Salinas, Ana María Ferreira

**Affiliations:** 1 Cátedra de Inmunología, Facultad de Ciencias/Facultad de Química, Universidad de la República (UdelaR), Montevideo, Uruguay; 2 Unidad de Biofísica de Proteínas, Instituto Pasteur de Montevideo, Montevideo, Uruguay; 3 Unidad de Bioquímica y Proteómica Analíticas, Instituto Pasteur de Montevideo, Montevideo, Uruguay; 4 Laboratorio de Carbohidratos y Glicoconjugados, Departamento de Química Orgánica/Departamento de Desarrollo Biotecnológico, Facultad de Química/Facultad de Medicina, Universidad de la República (UdelaR), Montevideo, Uruguay; McGill University, Canada

## Abstract

Antigen B (EgAgB) is the most abundant and immunogenic antigen produced by the larval stage (metacestode) of *Echinococcus granulosus*. It is a lipoprotein, the structure and function of which have not been completely elucidated. EgAgB apolipoprotein components have been well characterised; they share homology with a group of hydrophobic ligand binding proteins (HLBPs) present exclusively in cestode organisms, and consist of different isoforms of 8-kDa proteins encoded by a polymorphic multigene family comprising five subfamilies (*EgAgB1* to *EgAgB5*). *In vitro* studies have shown that EgAgB apolipoproteins are capable of binding fatty acids. However, the identity of the native lipid components of EgAgB remains unknown. The present work was aimed at characterising the lipid ligands bound to EgAgB *in vivo*. EgAgB was purified to homogeneity from hydatid cyst fluid and its lipid fraction was extracted using chloroform∶methanol mixtures. This fraction constituted approximately 40–50% of EgAgB total mass. High-performance thin layer chromatography revealed that the native lipid moiety of EgAgB consists of a variety of neutral (mainly triacylglycerides, sterols and sterol esters) and polar (mainly phosphatidylcholine) lipids. Gas-liquid chromatography analysis showed that 16∶0, 18∶0 and 18∶1(n-9) are the most abundant fatty acids in EgAgB. Furthermore, size exclusion chromatography coupled to light scattering demonstrated that EgAgB comprises a population of particles heterogeneous in size, with an average molecular mass of 229 kDa. Our results provide the first direct evidence of the nature of the hydrophobic ligands bound to EgAgB *in vivo* and indicate that the structure and composition of EgAgB lipoprotein particles are more complex than previously thought, resembling high density plasma lipoproteins. Results are discussed considering what is known on lipid metabolism in cestodes, and taken into account the *Echinococcus spp.* genomic information regarding both lipid metabolism and the EgAgB gene family.

## Author Summary

The larva of the cestode parasite *Echinococcus granulosus* affects a wide range of livestock mammals and humans, causing cystic echinococcosis (hydatid disease), a zoonosis with significant economic and public health impact. The disease is characterised by the growth of a fluid-filled cyst in the host's viscera (mainly liver and lung). The most relevant antigen for hydatid disease diagnosis is antigen B (EgAgB), a highly abundant lipoprotein present in the cyst fluid. There is overwhelming literature regarding EgAgB antigenicity and molecular characterisation at the protein and gene levels, but the knowledge of the lipids physiologically bound to EgAgB protein subunits is very scarce. Indeed, there is only one report showing that delipidated EgAgB binds fatty acids *in vitro*. This work describes the physiological lipids of EgAgB, an important piece of information to complete our knowledge on EgAgB molecular composition. In contrast to what was thought, EgAgB consists of a variety of neutral and polar lipid classes, associated to protein subunits, forming a plasma lipoprotein-like particle of 229 kDa in size. Taken into account that *E. granulosus* cannot synthesise fatty acids and sterols, these data suggest that EgAgB plays a role in the uptake and transportation of these essential lipids across parasite structures.

## Introduction

The larval stage of the cestode parasite *Echinococcus granulosus* is the causative agent of cystic echinococcosis (hydatid disease) in a range of mammalian species (mainly domestic ungulates) as well as in humans. It is a unilocular fluid-filled cyst, which steadily grows inside host visceras (mostly liver and lung). One of the major molecules produced in large amounts by the cyst is a highly immunogenic lipoprotein named antigen B (EgAgB) [1], [2], which represents a major diagnostic antigen for human infection [3]–[5]. This antigen is present in various larval locations including the parasite cellular layer of the cyst wall (germinal layer), the larval worms or protoscolex (asexually produced towards inside the cyst) and the hydatid cyst fluid (HCF). HCF is a complex mixture of parasite excretory-secretory products and host-derived molecules that constitutes the liquid content of the cyst [6]–[8]. Evidence for EgAgB presence in host circulation is very limited [9]. The strong antibody response mounted by infected patients against this antigen indicates that it is likely released into the host-parasite interface. However, it is unknown whether it is released throughout the infection or just at a certain time point [10], [11].

A lot of efforts have been made to understand the molecular composition/organization of EgAgB (reviewed by [12]). The native antigen is a lipoprotein which exhibits an estimated molecular weight of 120 to 160 kDa according to sedimentation equilibrium and gel filtration studies, respectively [1], [2]. The apolipoprotein components of EgAgB are encoded by a polymorphic multigene family that comprises five clades named *EgAgB1* to *EgAgB5* [13]–[17]. There is a long and yet unsettled controversy regarding EgAgB gene copy number. Based on the characterisation of *E. granulosus* isolates from different geographic origins, a recent study has proposed that there are at least 10 EgAgB distinct genes, including four and three different genes corresponding to the *EgAgB3* and *EgAgB4* clades [18]. However, a recent analysis of EgAgB loci in the current assembly of *E. granulosus* genome revealed the presence of seven EgAgB loci clustered on a discrete region of the genome, with one copy each of *EgAgB1*, *EgAgB2*, *EgAgB4* and *EgAgB5*, as well as three slightly differing copies of *EgAgB3* [19]. Outside this cluster only an EgAgB pseudogene was detected. However, the authors did not rule out the possibility of additional EgAgB genes in extra-chromosomal DNA arrays that might have slipped the genome assembly process [19]. There is evidence that EgAgB genes are differentially expressed in single life-cycle parasite stages, and also within distinct tissues of a same parasite stage (i.e. protoscolex and germinal layer) [18], suggesting that structural and/or functional differences between individual EgAgB lipoproteins may exist. The comparison of the amino acid sequences between members of EgAgB family showed that members of the EgAgB1, EgAgB3 and EgAgB5 clades are more similar among each other than to members of the EgAgB2 and EgAgB4 clades and vice versa [18]. The polypeptides encoded by these genes are between 65 and 71 amino acids long, and have approximately 8 kDa in mass; reason by which these apolipoproteins have traditionally been called EgAgB8/1 to EgAgB8/5. Some of them were found to be capable of self-associating into homo- or hetero-oligomers of 16 and 24 kDa [20] or even into higher order homo-oligomers [21].

Although EgAgB has been studied in some detail at the protein level, very little is known concerning its lipid moiety. EgAgB was originally described as a lipoprotein on the basis that lipids were non-covalently bound to the protein component since they could be mostly removed by extraction with alcohol/ether mixtures [2]. However, the characterisation of the lipid component has not been attempted; neither has the protein/lipid stoichiometry been determined nor the class lipid composition. More recently, it has been shown that EgAgB apolipoproteins belongs to a family of hydrophobic ligand binding proteins, referred to as HLBPs, found exclusively in cestode organisms. To date, members of this family include intracellular HLBP identified in *Monienza expansa* [22], [23] and *Hymenolepis diminuta* [24], [25] as well as extracellular HLBP identified as secreted components of *Taenia solium* and *Echinococcus granulosus* [26]. All these proteins have been found to be highly abundant and immunogenic, and exists as high-molecular-mass oligomers composed by α helix-rich subunits of about 7–11 kDa. Related immunogenic proteins were also described in *Taenia crassiceps* and *Taenia hydatigena* although their lipid-binding properties have not been analysed [26]–[29]. The ligand specificity of intracellular HLBPs has been characterised *in vitro* [22]–[25]; they bind saturated and unsaturated fatty acids (but not their CoA-ester derivatives), retinoids, and some antihelminthic drugs, and the *M. expansa* protein can also bind cholesterol. The *in vitro* lipid binding properties of extracellular HLBPs has been partially examined by binding assays using fluorescent lipid analogues and shown to bind fatty acids only [26], [30], [31]. In the case of EgAgB, the delipidated native molecule and the recombinant EgAgB8/1 and EgAgB8/2 apolipoproteins showed ability to bind a palmitic acid fluorescent analogue with high affinity, but the possibility that these proteins could bind lipids other than fatty acids was not evaluated [30].

Cestodes have a very restricted lipid metabolism. On the one hand, lipids are not suitable substrates for energy metabolism because they cannot be oxidised due to the limited aerobic capacity of tissue-dwelling parasites (reviewed by [32] and [33]). On the other hand, cestodes are unable to synthesise fatty acids, phospholipids and cholesterol *de novo*. Yet, lipids are required for biosynthetic purposes, and thus, parasite lipid-binding proteins play a key role in cestode metabolism, as they are likely involved in the uptake of lipids or their precursors from the host. In this scenario, it is generally thought that EgAgB and its secreted homolog could have an important role in the biology of cestodes, controlling the acquisition and distribution of lipids to specific tissues. Alternatively, it has been proposed that HLBPs could act as messenger molecules by carrying signalling lipids which would play a role in cell activation and/or differentiation processes involved in parasite adaptation to the host immune system. In the case of EgAgB, i*n vitro* evidence suggests that this lipoprotein may modulate host defenses by down-regulating neutrophils and dendritic cell-mediated innate responses as well as T-cell dependent mechanisms, which globally influence the intensity and quality of the adaptive immune responses [34]–[37].

The present work was aimed at identifying the native lipid moiety of EgAgB in order to complete our knowledge on the EgAgB molecular composition; this information could simultaneously shed light into EgAgB structure and function. Of particular relevance was to determine whether EgAgB binds *in vivo* lipid classes other than fatty acids. For that purpose, we purified EgAgB to homogeneity, using a protocol based on ion exchange chromatography coupled to immunoaffinity with a monoclonal antibody (Mo EB7), and then purified the EgAgB lipid moiety by extraction with organic solvents. Characterisation of immunopurified EgAgB of bovine origin showed that lipoprotein particles are constituted mostly by EgAgB8/1 apolipoprotein and that the native lipid moiety of this antigen comprises neutral and polar lipids that have not been previously described as ligands of this HLBP family.

## Materials and Methods

### Reagents

Inorganic salts, 3,5-di-*tert*-butyl-4-hydroxytoluene (BHT), ethylenediaminetetraacetic acid (EDTA) and authentic lipid standards including cholesterol (CH), fatty acids (FA), triacylglycerols (TAGs), phosphatidylethanolamine (PE), cardiolipin (CLP), phosphatidylinositol (PI), phosphatidylserine (PS) and phosphatidylcholine (PC) were acquired from Sigma Chemicals (USA). Solvents (HPLC grade or better) and α-naphthol were purchased from Merck (Germany) or Fisher Scientific (USA).

### Parasite material

*E. granulosus* HCFs from cysts containing protoscoleces of bovine origin were obtained by aspiration of the content of cysts present in lungs and livers of naturally infected cattle. Cysts were collected during the routine work of local abattoirs in Montevideo (Uruguay). *E. granulosus* HCFs of human origin, collected from surgically-removed hepatic hydatid cysts, were generously donated by Dr A. Leites and Dr E. Torterolo (Hospital Militar, Montevideo, Uruguay). All HCF samples were preserved by addition of 5 mM EDTA and 20 µM BHT, and maintained at −20°C. For EgAgB purification, three batches of bovine HCF (each one containing a pool of HCF from individual cysts) and two samples of individual human HCF were used.

### Purification of native EgAgB

Native EgAgB was purified from HCF following a previously described protocol [20] with slight modifications. HCF was centrifuged at 10000 *g* for 30 min at 4°C and the resulting supernatant filtered consecutively through 5, 2, 0.8 and 0.45 µm filter membranes (Millipore). The clarified HCF was firstly fractioned by anion exchange chromatography on a Q-Sepharose column (Pharmacia Biotech, Uppsala, Sweden) previously equilibrated in 20 mM phosphate buffer, pH 7.2 containing 200 mM NaCl, 5 mM EDTA and 20 µM BHT. After washing in equilibration buffer, the retained material was eluted by changing ionic strength to 400 mM NaCl in a single step. The eluted fraction (enriched in EgAgB and almost free of host albumin and immunoglobulins) was used to purify the antigen to homogeneity by immunoaffinity chromatography based on the utilization of a monoclonal antibody (MoAb) -named EB7- that specifically recognises the native lipoprotein [20]. For this purpose, the Q-Sepharose eluted fraction was diluted in 20 mM phosphate buffer, pH 7.2 containing 5 mM EDTA and 20 µM BHT, to reach a final concentration of 200 mM NaCl and then applied to the EB7-Sepharose column. After washing, EgAgB was eluted with 100 mM glycine-HCl, pH 3, immediately neutralised with 2 M Tris pH 9.6 and then equilibrated in 20 mM phosphate buffer, pH 7.2 containing 5 mM EDTA and 20 µM BHT (PBS-EDTA-BHT) using a PD-10 desalting column (Amershan, Biosciences). The homogeneity of EB7-affinity purified EgAgB (immunopurified EgAgB) was monitored by SDS-PAGE on 15% polyacrylamide gels followed by silver stain. In addition, samples were analysed by two-dimensional gel electrophoresis as described below.

### Identification of EgAgB apolipoproteins by two dimensional (2-D) gel electrophoresis and mass spectrometry analysis

First dimension was performed with commercially available IPG-strips (7 cm, linear 3–10, GE Healthcare). Immunopurified EgAgB was prepared and concentrated by using the 2-D Clean-Up kit (GE Healthcare) and dissolved in rehydration solution (7 M urea, 2 M thiourea, 2% CHAPS, 0.5% IPG buffer 3–10 (GE Healthcare), 0.002% bromophenol blue, DTT 17 mM). Samples in rehydration solution were loaded onto IPG-strips by passive rehydration during 12 h at room temperature. The isoelectric focusing was done in an IPGphor Unit (Pharmacia Biotech) employing the following voltage profile: constant phase of 300 V for 30 min; linear increase to 1000 V in 30 min; linear increase to 5000 V in 80 min and a final constant phase of 5000 V to reach total of 6.5 kVh. Prior running the second dimension, IPG-strips were reduced for 15 min in equilibration buffer (6 M urea, 75 mM Tris–HCl pH 8.8, 29.3% glycerol, 2% SDS, 0.002% bromophenol blue) supplemented with DTT (10 mg/ml) and subsequently alkylated for 15 min in same equilibration buffer but supplemented with iodoacetamide (25 mg/ml). The second-dimensional separation (SDS-PAGE) was performed in 15% polyacrilamyde gels using a SE 260 mini-vertical gel electrophoresis unit (GE Healthcare). The molecular size markers used were Amersham Low Molecular Weight Calibration Kit for SDS Electrophoresis (GE Healthcare). The gels were silver stained according to [38]. Images were digitalised using a UMAX Power-Look 1120 scanner and LabScan 5.0 software (GE Healthcare).

Identification of protein spots was performed by mass spectrometry (MS) using a 4800 MALDI TOF/TOF™ (AB Sciex). Briefly, peptide mass fingerprinting plus MS/MS ion search of selected spots were carried out by in-gel trypsin treatment (sequencing-grade, Promega) at 37°C, overnight. Peptides were extracted from gels using 60% acetonitrile in 0.1% TFA, concentrated by vacuum drying. Peptides were further concentrated and desalted using C18 reverse phase micro-columns (OMIX Pippete tips, Varian). Peptide elution from micro-column was performed directly into the mass spectrometer sample plate with 2 µl of matrix solution (α-cyano-4-hydroxycinnamic acid in 60% aqueous acetonitrile containing 0.1% TFA). Mass spectra of digestion mixtures were acquired in the MALDI-TOF/TOF mass spectrometer using the reflector mode. Spectra were externally calibrated using a mixture of peptide standards (Mix 1, AB Sciex). For increased confidence of identification, selected peptides were further fragmented by post-source decay (PSD) and collisional-induced dissociation (CID). Proteins were identified by NCBI nr database searching using the MASCOT program (Matrix Science http://www.matrixscience.com/search_form_select.html) and using the following search parameters: monoisotopic mass tolerance, 0.05 Da; fragment mass tolerance, 0.2 Da; carbamidomethyl cysteine and methionine oxidation as variable modifications and up to one missed tryptic cleavage allowed.

### Lipid extraction and purification

Total lipids were extracted according to the methodology described by [39], with slight modifications. Briefly, HCF (previously concentrated 10-times using a Savant SpeedVac System) or immunopurified EgAgB (1 mg protein in approximately 2 mL of PBS-EDTA-BHT) were mixed with 30 ml of a CHCl_3_∶CH_3_OH mixture (2∶1) and vigorously shaken for 2 minutes. Next, the homogenate was filtrated and the extract washed with NaCl solution to reach a final concentration of 0.73% and a CHCl_3_∶CH_3_OH∶H_2_O ratio of 2∶1∶0.2 in volume. After vigorous agitation for 1 minute, the separation of phases was achieved by centrifugation at 2400 rpm for 20 min and the upper phase was removed by aspiration and discarded. The lower phase containing lipids was recovered and taken to dryness by rotary evaporation at 40°C under vacuum; last traces of solvent were removed by a stream of N_2_ (g). Finally, the lipid fraction was dissolved in CHCl_3_ to a concentration of 10 mg/mL. As a control of lipid contaminants in buffers and/or solvents, an equal volume of PBS-EDTA-BHT was used in parallel for extraction. Purified lipid extracts were stored at −20°C under N_2_(g) until analysis.

### Quantitation of total protein and lipid content

The protein content of immunopurified EgAgB preparations was determined using bicinchoninic acid in a microtitre plate assay (BCA Protein Assay kit) with BSA as standard (Pierce, Rockford, Ill.). Total lipids in EgAgB were determined gravimetrically by weighting purified lipids immediately after extraction and solvent removal under a stream of N_2_(g).

### Qualitative and quantitative analysis of lipid extracts

Separation of lipid class components was performed by high-performance thin layer chromatography (HPTLC) on Kieselgel 60 plates (Merck). HPTLC plates (10×10 cm) were pre-washed by migration in CHCl_3_/CH_3_OH (2∶1 v/v) and then activated at 100°C for 30 min. Lipid samples (fractions obtained from HCF and EgAgB and standards) were spotted manually using a micro-syringe (Hamilton). Double development was initially carried out as follows: plates were first half-developed using a mobile phase for resolving polar lipids (PL), and after drying under a N_2_ (g) stream, were developed to completion in a mobile phase for neutral lipids (NL). Single development for resolving PL or NL was also performed. The solvent systems used as mobile phase were: for PL resolution- methyl acetate/isopropanol/chloroform/methanol/0.25%KCl (25∶25∶25∶10∶9, v/v/v/v/v) and for NL resolution- hexane/diethyl-ether/acetic acid (80∶20∶1, v/v/v). Lipid bands were visualised by spraying the plates with 8% (m/v) CuSO_4_ in a 10% (v/v) H_3_PO_4_ aqueous solution, and heating at 140°C; the identification of lipid classes was performed by comparison with primary and secondary standards run on the same HPTLC plate. The relative abundance of each lipid class respect to the total lipid content could not be estimated because not all lipids were resolved adequately in a single HPTLC using double development. We estimated the percentage of individual lipid classes in the total of NL or PL instead. 1-nonadecanol was used as internal standard for normalization. HPTLC plates were scanned and the intensity of the bands was determined using the ImageJ software (http://rsb.info.nih.gov/ij/). In addition, specific staining for sterol esters/sterols and glycolipids were carried out using FeCl_3_.6H_2_O and α-naphthol, respectively [40]. For the latter analysis a lipid fraction from murine macrophage-like J774.A1 cells was prepared to use as a control. Briefly, J774.A1 cells (generously donated by Dr. M. Noel Alvarez, Departamento de Bioquímica, Facultad de Medicina, UdelaR) were washed with PBS, lysed using a hypotonic solution (0.25 mM phosphate 3.75 mM NaCl) and centrifuged (15000×*g*, 4°C for 30 min) to obtain a cell membrane enriched fraction. Lipids were then extracted following the Folch method as described above.

### Fatty acid analysis by gas-liquid chromatography (GC)

The fatty acid composition of EgAgB and HCF lipid fractions was analysed by gas-liquid chromatography of their methyl esters derivatives (FAMEs); for these studies material of both human and bovine origin were used. Lipid fractions were subjected to acid methanolysis with 1% H_2_SO_4_ in methanol at 50°C for 16 h. The purified FAMEs were dissolved in hexane and then subjected to GLC analysis on a Hewlett-Packard 5890 equipped with a Carbowax 20 capillary column and flame ionisation detector (FID). The oven temperature was initially set at 180°C for 10 min, and then increased at 2.5°C/min to 212°C, level at which was held for 10 min. The individual FAMEs peaks were identified by comparison of their retention times with those of authentic FAMEs standards.

### Determination of the molecular weight of native immunopurified-EgAgB by Size-Exclusion Chromatography with on-line Multiangle Light-Scattering (SEC-MALLS)

Light scattering analysis was carried out using a Superset 200 HR 10/30 SEC column (Amersham Biosciences, Piscataway, NJ), connected to an HPLC system (Schimatzu) at room temperature. Immunopurified EgAgB (200 µL of a 0.7 mg/mL solution in PBS) was applied onto the column previously equilibrated in PBS, and elution was monitored with on-line detection using the following detectors: multiangle laser light scattering (miniDAWN system, Wyatt Technology Corporation, Santa Barbara, CA), ultraviolet UV (SPD-20A, Shimadzu) and differential refractive index (RID-10A, Shimadzu). In addition, plasma-derived high density lipoprotein (HDL) was analysed in the same conditions for comparison. For HDL preparation, human plasma was obtained from a healthy donor and HDL purification was carried out following conventional ultracentrifugation methods [41]. A written consent was obtained from the donor according to the Ethic Committee of the Faculty of Chemistry (UdelaR) and the Executive Decree N° 379/008. Light scattering data were collected and processed with ASTRA software (v4.73.04, Wyatt Technology) using the Debye fit method with a *dn/dc* ratio set to 0.186 mL/g [42].

## Results

### EgAgB8/1 is the major apolipoprotein component of EgAgB immunopurified from bovine HCF

**Figure 1:**
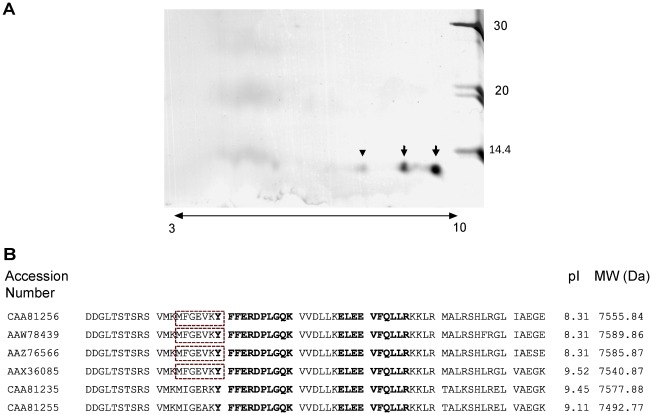
EgAgB8/1 is the major apolipoprotein component of EgAgB immunopurified from bovine HCF. A) Immunopurified EgAgB of bovine origin was analysed by 2-D gel electrophoresis using a 3–10 lineal gradient of pH in the first dimension, 15% acrylamide gel for SDS-PAGE and silver staining for development. Two main spots focused at pH of 9.6 and 8.8 are observed (arrows); a minor component focused at pH 7.9 is also indicated (head arrow). Identification of spots observed in A) was carried out by mass spectrometry as detailed in 2.4. Panel B) shows the amino acid sequences corresponding to various isoforms described for EgAgB8/1 in which are indicated: the main peptides found in all spots (in bold) and a peptide detected only from the analysis of the more basic spot (within a box). The theoretical isoelectric point (pI) and molecular weight (MW) of each EgAgB8/1 isoform are shown (determined using Compute pI/MW Expasy tool at http://web.expasy.org/compute_pi/).

Most studies were performed with EgAgB of bovine origin since the availability of parasite material of human origin was very limited. EgAgB was purified to homogeneity from bovine HCF using a previously described procedure based on selective adsorption of this antigen on Q-Sepharose followed by immunoaffinity chromatography using immobilised MoAb EB7 [20]. This purification methodology was found to be suitable for the main objectives of this work, as it has been shown that affinity immunoadsorption permits isolation of lipoproteins under minimally perturbing conditions [43]. We characterised the apolipoprotein component of bovine EgAgB using 2-D gel electrophoresis and found that it contained two major spots electrofocused at pH 8.8 and 9.6 (Figure 1A, arrows). These two spots were identified as EgAgB8/1 by mass spectrometry analysis. Identification was mainly due to the analysis of the data arising from two main peptide signals with *m/z* of 1275.68 and 1399.69 that matched with the ELEEVFQLLR and YFFERDPLGQK sequences, respectively. These peptides lie in conserved regions of the amino acid sequence of EgAgB8/1. Therefore, we cannot discriminate which ones of the already described EgAgB8/1 isoforms is present in the samples. These isoforms include molecules having different theoretical isoelectric point (Figure 1B). In the case of the most basic spot, focused at pH 9.6 (Figure 1A, arrow), an additional peptide matching the MFGEVK sequence (Figure 1B, dashed line box) was observed. This sequence is present in at least four EgAgB8/1 isoforms. Interestingly, only one of them has a theoretical pI of 9.52, matching the observed value (Figure 1B, solid line box). A minor spot electrofocused at pH 7.9 was also detected (Figure 1A, head arrow), and peptide mass spectrometry of this spot indicated that it also corresponded to EgAgB8/1. When higher amounts of antigen (3-fold) were analysed, EgAgB8/4 was detected; identification was mainly based on the presence of a signal with *m/z* = 1152 that matched with the LGEIRDFFR sequence, which is only present in EgAgB8/4 isoform (data not shown).

### The lipid component of immunopurified EgAgB is highly heterogeneous, containing a variety of neutral and polar lipid classes

Previous work suggested that EgAgB8 apolipoproteins are capable of binding fatty acids [30]. However, the fact that HCF, the physiological milieu to which EgAgB is secreted, contains a wide range of neutral and polar lipids [44], [45], opens up the possibility that the putative physiological ligands of EgAgB8 apolipoproteins include a more diverse set of lipids. For identification of these ligands, we firstly purified the lipid fractions of both, EgAgB and the HCF from which this antigen was immunopurified, using parasite material of bovine origin via the Folch extraction method. This procedure is broadly applied to the analysis of lipoproteins and has the advantage of solubilising all major lipid classes using a single solvent mixture, even though it does not allow the protein to be recovered because this fraction irreversibly precipitates during the procedure. From the dry mass of total lipids extracted and the protein concentration of the starting sample we estimated the lipid∶protein ratio (w∶w), finding that it was plainly higher for immunopurified EgAgB (between 0.6∶1 and 1.1∶1, n = 3 independent batches) than HCF (between 0.17∶1 and 0.19∶1, n = 3 independent batches). This implies that the lipid fraction of EgAgB represented approximately 40–50% of its total mass.

**Figure 2:**
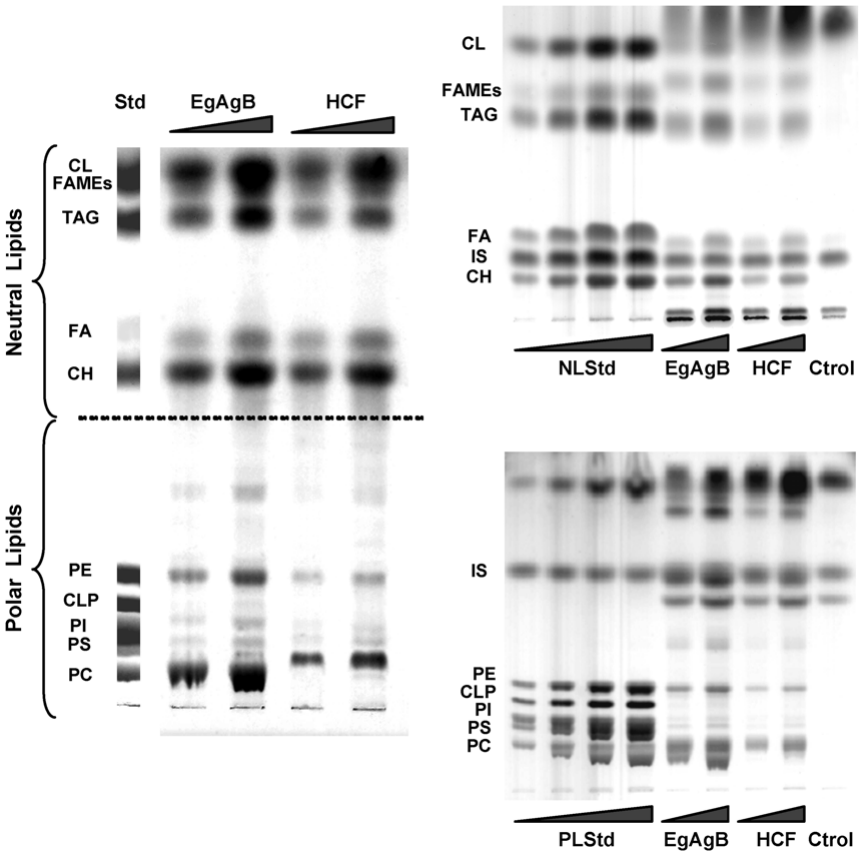
The lipid fractions of EgAgB and HCF are similar and include a variety of neutral and polar lipids. The lipid fractions of bovine EgAgB and its corresponding HCF were analysed by HPTLC using double development (A) or in conditions for resolving separately neutral (B) and polar (C) lipid classes. Standards (between 2 and 8 µg) and samples (around 5 and 10 µg) were applied onto HPTLC plates; 1-nonadecanol was added as internal standard (IS) for normalization purposes. Lipid bands were visualised using CuSO_4_/H_3_PO_4_ and identified by comparison with the standards. The profile is representative of three EgAgB and HCF batches, which were independently analysed. The wedges indicate increasing amount of loading samples. NLStd: neutral lipid standard; CH: cholesterol; FA: free fatty acid; TAG: triacylglycerols; FAMEs: fatty acid methyl esters; CL: cholesteryl laurate; PLStd: polar lipid standard; PC: phosphatidylcholine; PS: phosphatidylserine; PI: phosphatidylinositol; CLP: cardiolipin and PE: phsophatidylethanolamine.

As an initial approach for examining the complexity of lipid classes, HCF and EgAgB lipids were analysed by HPTLC using double development. Under these conditions the majority of neutral and polar lipids are clearly separated, although the resolution of some lipid classes may not be optimal. By analysing in parallel a mixture of authentic lipid standards, we observed that the natively-bound lipid component of immunopurified EgAgB is highly heterogeneous, comprising several lipid classes, all of which are also found in the HCF. Indeed, the lipid fractions of both EgAgB and HCF showed to contain a wide variety of lipid classes from very hydrophobic ones (compatible with sterol esters and TAGs) to charged ones (phospholipids) (Figure 2A); this pattern was obtained for EgAgB and HCF samples derived from three independent batches (data not shown). Thus, these results indicated that the lipid fraction of EgAgB particles carrying EgAgB8/1 apolipoproteins consists not solely of fatty acids, but also of a variety of polar and neutral lipids present in HCF. For comparative purposes, we analysed the lipid fraction of EgAgB immunopurified from HCF of human origin (two independent batches), finding similar results in terms of lipid∶protein ratio as well as lipid class composition (data not shown).

**Figure 3:**
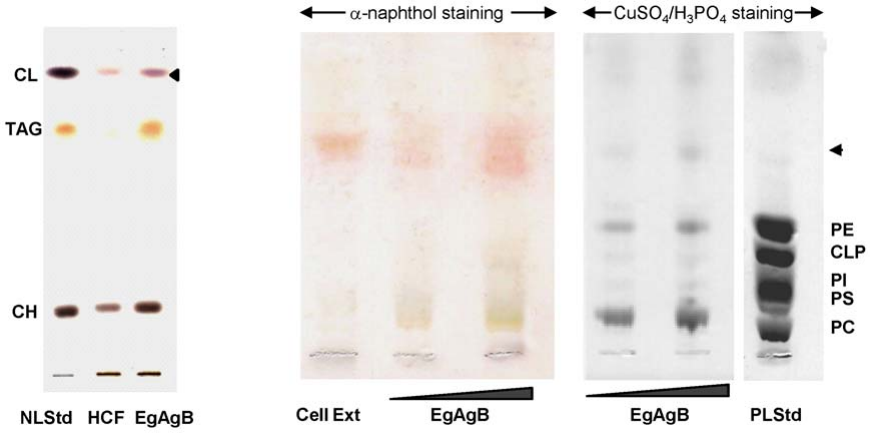
Immunopurified EgAgB lipid moiety include sterols, sterol esters and glycolipids. (A) The lipid fractions (around 10 µg) of bovine EgAgB and its corresponding HCF were applied on HPTLC plates and chromatographed using a solvent system for resolving neutral lipids. Development was performed using an acidic ferric chloride solution for specific staining of sterols/sterol esters as violet bands. NLStd, containing cholesterol, cholesteryl laurate, triacylglycerides and free fatty acids, was analysed in parallel as a control. (B) The lipid fraction (around 2 and 4 µg) of bovine EgAgB was applied on HPTLC plates and chromatographed using a solvent system for resolving polar lipids. Development was carried out using both, a general (CuSO_4_/H_3_PO_4_) and a glycolipid specific stain employing α-naphthol, which yields glycolipids as purple bands. As a control a cell membrane extract (Cell Ext) was analysed in parallel. The wedges indicate increasing amount of loading samples. CH: cholesterol; TAG: triacylglycerols; CL: cholesteryl laurate; PC: phosphatidylcholine; PS: phosphatidylserine; PI: phosphatidylinositol; CLP: cardiolipin; PE: phosphatidylethanolamine.

In order to improve the identification of the lipid classes of EgAgB, the EgAgB lipid fraction was analysed by HPTLC using conditions for resolving separately neutral and polar lipids; in this analysis, 1-nonadecanol was added to all samples as an internal standard (IS) for normalization. This analysis was carried out only for HCF and EgAgB of bovine origin. As shown in Figures 2B and 2C, the lipid composition of EgAgB and HCF was very similar in terms of the variety of lipid classes. Among neutral lipids, three classes were assigned considering their mobility in comparison with standards: TAGs, free sterols and free fatty acids. In addition, three components having higher mobility than TAGs were observed in both EgAgB and HCF lipid fractions. Among these, there was a component that migrated slightly faster than FAMEs but slower than cholesteryl laureate, being compatible with dialkyl-monoacylglycerols and/or alkenyl-diacylaglycerols [40]. The other two components were not completely resolved and migrated as a wide smear in the assay conditions. The least mobile component of this smear could correspond to sterol esters according to the mobility of cholesteryl laurate. This was confirmed by using a sterol ester-specific staining method based on the formation of a purple complex with FeCl_3_ at acid pH (Figure 3A). The most mobile component was also observed in the control, indicating that it corresponded to a very hydrophobic contaminant derived from the extraction procedure. A second contaminant having a very low mobility in the assayed conditions was also detected in the control.

With respect to polar lipids, the analysis of EgAgB and HCF in parallel with phospholipid standards showed that phosphatidylcholine was the main phospholipid present in both samples. Phosphatidylethanolamine, phosphatidylinositol and phosphatidylserine were also detected in smaller amounts as well as traces of cardiolipin. Furthermore, minor components with higher mobility than phospholipids but lower than neutral lipids compatible with glycolipids were observed. The presence of glycolipids was confirmed by staining with α-naphthol, a dye that specifically reacts with sugar groups (Figure 3B).

**Figure 4:**
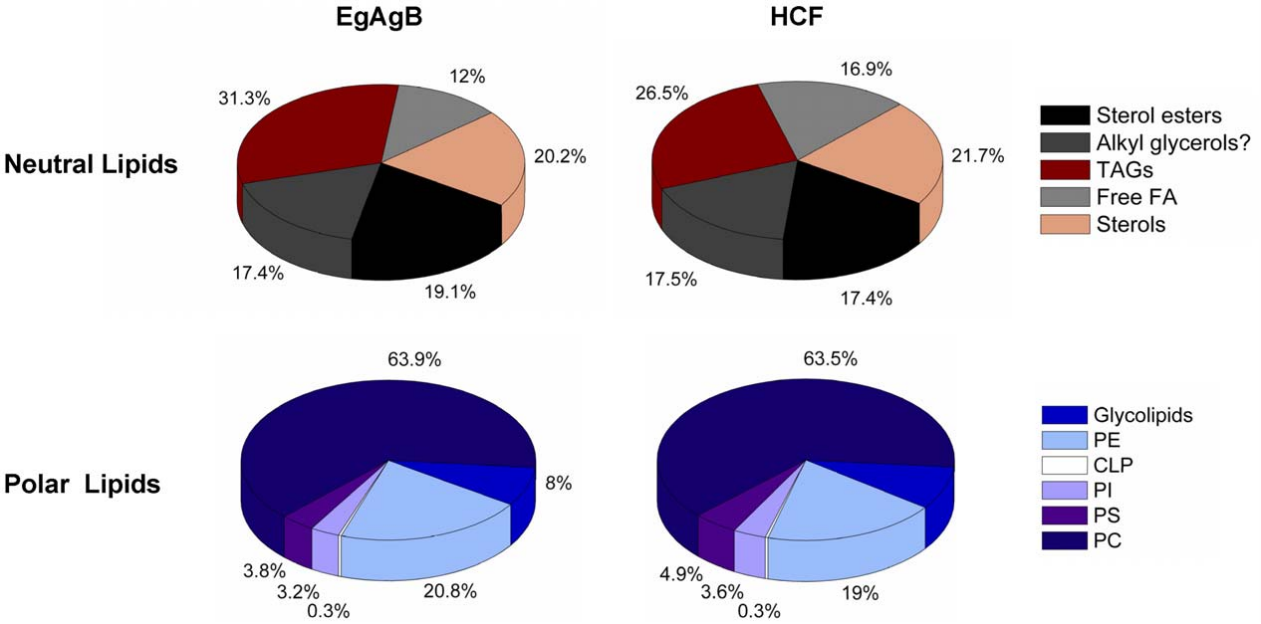
Relative abundance of the main lipid classes present in EgAgB and HCF. EgAgB and HCF of bovine origin were separately analysed by HPTLC in conditions to resolve neutral or polar lipids. The percentage of each lipid class in the total of neutral or polar lipids was estimated by densitometry. Results are expressed as mean values obtained for three different batches of EgAgB and HCF, which were analysed in duplicates. The question mark corresponds to a lipid class whose identity is based solely on its HPTLC mobility. CH: cholesterol; TAG: triacylglycerols; CL: cholesteryl laurate; PC: phosphatidylcholine; PS: phosphatidylserine; PI: phosphatidylinositol; CLP: cardiolipin; PE: phosphatidylethanolamine.

The relative abundance of the major lipid classes found in immunopurified EgAgB and HCF within total neutral or polar lipids was estimated as shown in Figure 4. This estimation was carried out by analysing the intensity of HPTLC bands by densitometry, for which errors due to unequal sample application or irregular staining across the plate were normalised using the internal standard. TAGs and phosphatidylcholine corresponded to the most abundant neutral and polar lipids of EgAgB, reaching around 30% and 60%, respectively; similar percentages of these lipids were found in HCF. Moreover, the relative abundance of any lipid class within neutral and polar lipids was almost identical for EgAgB and HCF, suggesting a non-selective binding of lipids by EgAgB8/1 apolipoproteins. It is worth to mention that the relative abundance values are likely affected by two factors inherent to the method: i) the fact that the intensity of staining does not follow the same proportionality with the lipid mass for the different lipid classes, and ii) the spreading of the band affects densitometry, which means that how each lipid is resolved/focused during the chromatography could influence its quantitation.

### The main fatty acids present in immunopurified EgAgB are 16∶0, 18∶0 and 18∶1(n-9)

**Figure 5:**
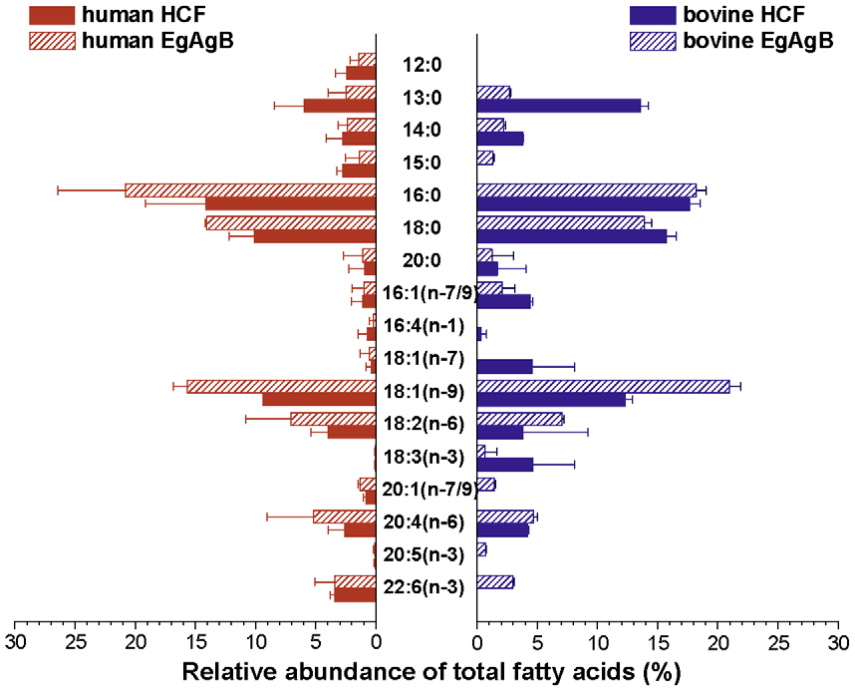
Relative abundance of total fatty acids of immunopurified EgAgB and HCF lipid fractions. The methyl ester derivatives of total (free and esterified) fatty acids (FAMEs) were prepared from EgAgB and HCF lipid fractions and then analysed by GLC. The graphic shows the relative abundance of identified components calculated as the percentage of each fatty acid referred to the total content. In the cases where the resolution of two components was partial, their abundances were taken together (i.e. 16∶1(n-7/9) refers to the content of 16∶1(n-7) y 16∶1(n-9)). The values correspond to the mean ± std of two batches of EgAgB and HCF of bovine and human origin.

We next analysed the fatty acid composition of total lipids extracted from human and bovine EgAgB and HCF (Figure 5). Globally, the results revealed that the fatty acid profiles of EgAgB obtained from both sources were very similar. The predominant fatty acids were 16∶0, 18∶0 and 18∶1(n-9), while 18∶2(n-6) and 20∶4(n-6) were present in a much lower proportion. Other fatty acids were present in minor quantities, representing less than 5% each. Furthermore, the content of fatty acids in EgAgB closely resembles that of the HCF used for its purification, suggesting that EgAgB8/1 apolipoproteins are capable of binding the most abundant fatty acids of HCF in a non-selective manner. Nevertheless, EgAgB8/1 apolipoproteins showed some degree of selectivity in fatty acid binding properties since the relative abundance of 13∶0, 16∶0, 18∶0 and 18∶1(n-9) were similar in bovine HCF, but not in bovine EgAgB, which contained lower percentage of 13∶0 and higher percentage of 18∶1(n-9) than bovine HCF. The comparison of the relative abundance of fatty acids in bovine vs. human HCF strongly suggests that the fatty acids are taken from the host, since 13∶0, a more abundant fatty acid in ruminants, was found in higher levels in bovine than human HCF (14 vs. 3% of total fatty acids).

### Native EgAgB comprises a heterogeneous population of particles having a size distribution similar to HDL

**Figure 6:**
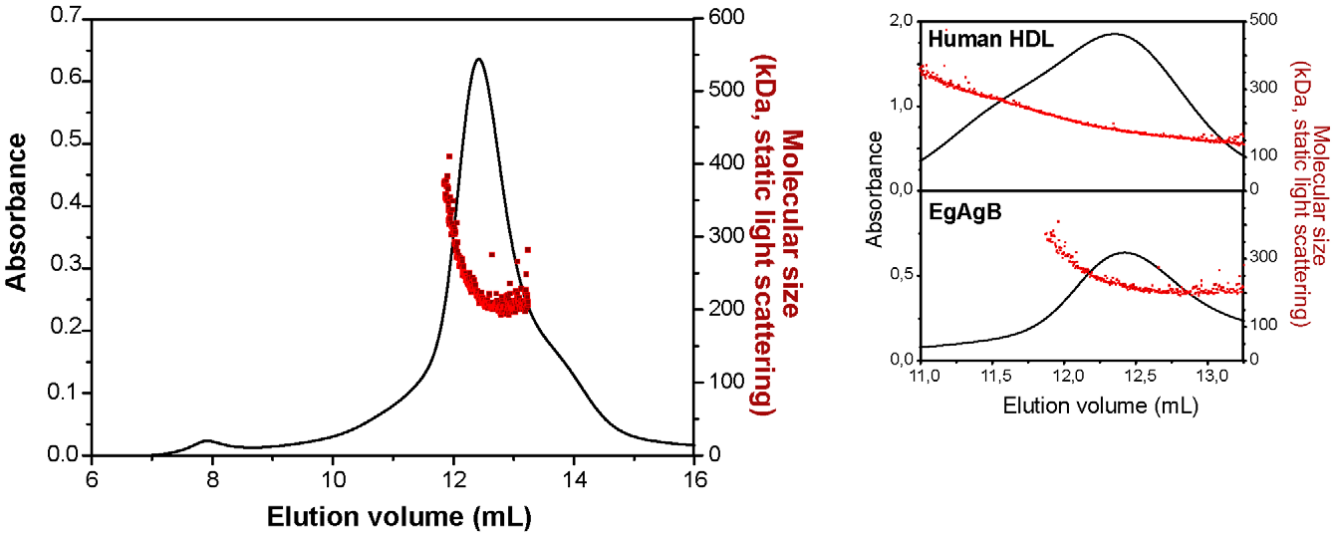
Solution behaviour of native EgAgB characterised by SEC-MALLS. Representative SEC-MALLS analysis of native EgAgB after immunopurification from bovine HCF. The solid curve represents the UV absorbance profile at 280 nm, overlaid with the scatter plot, which corresponds to the mass estimated from light scattering profile and concentration measurement, versus the elution time. The calculated molecular masses are given in the text. The *inset* shows the MALLS analysis of human HDL (top), and that of bovine EgAgB (bottom) for comparison.

Bovine EgAgB was analysed by SEC to study its physical state in aqueous solution. EgAgB eluted as a main peak centered at 12.5 mL, from which an apparent MW of 160 kDa was estimated by comparison with protein standards. This value is in agreement with previous results [1], [2]. In order to determine the absolute MW independently of hydrodynamic assumptions, we also analysed the MW of bovine EgAgB by SEC-MALLS. The molecular mass curve obtained by SEC-MALLS displayed two phases (Figure 6): an initial sharp decrease of the size at the beginning of elution (started at molecular-mass species of ∼400 kDa), rapidly followed by a plateau at a calculated mass of ∼229 kDa, which included the maximum and extended to the end of the peak. This behaviour suggests that EgAgB exists in solution as a heterogeneous population of lipoprotein particles, with most species showing an average molecular mass of 229±7 kDa. This heterogeneity could be, at least partially, associated to the capacity of EgAgB apolipoproteins to form particles accommodating variable amounts of lipids. Another possibility is that the lipoprotein could have some tendency to self-associate, leading to the formation of (less abundant) higher-order oligomers. On the other hand, the lipid/protein mass ratio, lipid composition and size of EgAgB suggested that, globally, it exhibits compositional and dimensional characteristics resembling those of plasma HDL. For comparison, human HDL was then analysed by SEC-MALLS under the same conditions. HDL showed a similar SEC-chromatographic profile to EgAgB, exhibiting molecular mass species from 150 to 400 kDa, with a mean MW of 209±4 kDa (Figure 6, *inset*).

## Discussion

This work aimed at characterising the native lipid moiety of EgAgB. As we have already mentioned, studies were mainly performed with EgAgB of bovine origin, yielding data that improve our knowledge on EgAgB lipoprotein composition. In addition, the characterisation of EgAgB protein∶lipid ratio and EgAgB lipid composition (major lipid classes and total fatty acids) was carried out with limited amounts of human EgAgB, obtaining similar results that confirm our findings.

EgAgB was purified from HCF using a previously described method based on anion exchange followed by immunoaffinity on MoAb EB7-Sepharose [20]. For a complete description of the lipoprotein composition we firstly characterised the apoliprotein component of immunopurified EgAgB of bovine origin. In addition, the identification of the apolipoprotein subunits forming EgAgB is relevant because the lipid-binding properties of EgAgB8 isoforms may differ. Native as well as recombinant EgAgB8/1 and EgAgB8/2 did not show differences in their capacity to bind fatty acid analogues *in vitro* [30]. Nevertheless, the physiologic lipid ligands of EgAgB8 isoforms could depend not only on the lipid-binding properties of individual isoforms, but also on the cell/tissue compartment where they are synthesised, assembled and/or transported (e.g. germinal layer vs. protoscolex). We found that EgAgB8/1 was the major apolipoprotein component of immunopurified EgAgB (Figure 1), while EgAgB8/4 was detected in much smaller amounts. In the original article describing this purification method, peptides matching sequences of EgAgB8/1, EgAgB8/2 and EgAgB8/4 were found, although those corresponding to EgAgB8/4 (SDPLGQK and LGEIR) were not initially assigned to this molecule, because its sequence was unknown. The purification of lipoproteins carrying EgAgB8/1 agrees with the fact that EgAgB8/1 is expected to be present in HCF of bovine origin [46] and MoAb EB7 strongly reacts with this isoform [20]. In contrast, MoAb EB7 does not bind to EgAgB8/2 [20] and it is unlikely that it binds to EgAgB8/4 since this isoform has much higher similarity to EgAgB8/2 than to EgAgB8/1 [18]. Therefore, purification by this method of particles carrying EgAgB8/2 and/or EgAgb8/4 apolipoproteins may result from lipoprotein particles carrying EgAgB8/1 along with EgAgB8/4 and/or EgAgB8/2, and/or associative interactions between lipoprotein particles carrying different EgAgB8 isoforms. In this regard, it is worthy to note that association of EgAgB particles could occur according to the EgAgB analysis by SEC-MALLS (Figure 6) and previous observations made by studying EgAgB sedimentation equilibrium [2]. The fact that we did not detect EgAgB8/2 in immunopurified EgAgB of bovine origin may be due to the sensitivity of the technique or to variations in the composition of distinct HCF pools. Indeed, the presence of EgAgB8/2 was detected in bovine HCF by Chemale *et al.* [30] but not by Aziz *et al.* [46] and it seems to be more variable than that of EgAgB8/1 and EgAgB8/4 among cysts of different origin/fertility [46]. Furthermore, EgAgB8/2 would not be one of the most abundant EgAgB isoforms in the HCF according to the expression levels of EgAgB family in *E. granulosus* metacestode [18]. Therefore, the characterisation of the apolipoprotein composition of EgAgB highlights that HCF-derived EgAgB preparations may differ depending on parasite material and these differences may be relevant when analysing EgAgB structural and/or functional properties. In any case, since *EgAgB8/1* is highly expressed in the *E. granulosus* metacestode [18], [19], the EgAgB8/1-enriched lipoprotein that we used for lipid characterisation likely represents one of the most abundant lipoproteins of the EgAgB family present in HCF.

Analysis of the lipid composition of immunopurified EgAgB of both bovine and human origin, revealed a high diversity in terms of lipid classes ranging from highly hydrophobic lipids (mainly TAG, but also sterol esters) to a variety of phospholipids (mainly phosphatidylcholine). These findings indicate that EgAgB is a more complex lipoprotein than previously suggested from lipid-binding studies *in vitro*, in which fatty acids were described as the main lipid ligands of EgAgB8/1 and EgAgB8/2 [30]. Also, these results set EgAgB apart from the fatty acid binding protein family, whose members bind mainly fatty acids [47]. On the other hand, the fact that the lipid moiety of immunopurified EgAgB represented between 40 and 50% of the total mass reveals that EgAgB require to adopt a very well organised structure to accommodate a high proportion of lipid molecules in a single particle, suggesting similarities with animal lipoproteins found in both invertebrate hemolymph and vertebrate plasma [48]. The structural organization of these animal lipoproteins is well established; the most hydrophobic lipids (triacylglycerols, cholesteryl esters and other lipid-soluble components) are sequestrated in a central core, surrounded by an external hydrophilic shell that contains the apolipoproteins and amphipathic lipids (mostly phospholipids and unesterified cholesterol) (review by [49]). A structure like this could explain the heterogeneity of molecular mass species observed during analysis of immunopurified EgAgB by SEC-MALLS. Among vertebrate plasma lipoproteins, EgAgB would be more similar to the smallest HDL particles, referred to as HDL_3_ [48], [50], which exhibits a lipid∶protein mass ratio (w∶w) between 0.67∶1 and 1.2∶1 (0,6∶1–1.1∶1 for EgAgB) and an average molecular mass of 200 kDa (229 kDa for EgAgB); the comparative analysis of HDL and EgAgB by SEC-MALLS supports this hypothesis (Figure 6, *inset*). However, the content of TAG in EgAgB is much higher than in HDL_3_, which likely reflects differences in the lipid transport function of these lipoproteins. In addition, in the context of this structural organisation share by all plasma lipoproteins, and taken into account its size and chemical composition, each EgAgB particle would contain between 11 and 15 EgAgB8 apolipoprotein molecules inserted into the outer phospholipid monolayer. This new scenario is relevant when considering the biological effects of EgAgB through parasite's as well as host's receptors. The exposure of more than a dozen of apolipoproteins on the surface of the lipoprotein particle would facilitate the establishment of multiple interactions with receptors, increasing the avidity of the interaction and the signals derived from it. The formation of multiple EgAgB-B cell interactions likely contributes to the immunogenicity of this lipoprotein. On the other hand, it cannot be ruled out that lipids could participate, at least partially, in some of the EgAgB biological activities (for example those anti-inflammatory described *in vitro*). Surface charge and/or hydrophobic distribution resulting from a lipoprotein ensemble (as opposed to lipid or protein fractions alone) may alter the type of receptors involved, affecting its physiological effects.

The identification of the native lipid ligands of EgAgB provides relevant information for studying the function of this HLBP family member. Evidence for HLBP-mediated fatty acid binding and transportation across parasite membrane has been obtained in *Taenia solium* [26]. Our results suggest that EgAgB apolipoproteins are likely involved in solubilising and stabilising a variety of insoluble lipids in a lipoprotein particle, and that this may have an important role to deliver lipids from the tissues/sites where are synthesised/sequestered, to those that utilise or storage them. The fact that the lipids present in EgAgB are not only fatty acids, but also other essential building blocks such as sterols, highlights that EgAgB could serve a role for the *E. granulosus* metabolic demand of lipids. In this context is important to highlight that no enzymes for either fatty acid anabolism or squalene synthesis (the precursor of the whole family of animal sterols) have been found in the *E. granulosus* transcriptome (data base http://www.compsysbio.org/partigene/) or the *Echinococcus multilocularis* genome (unpublished observations, http://www.genedb.org/Homepage/Emultilocularis). In addition, metabolic studies have demonstrated that sterol synthesis in *E. granulosus* seems to stop at the level of farnesyl or nerolidol pyrophosphate and that the content of cholesterol in HCF derives from the cholesterol pool of the host [51], [52]. Thus, *E. granulosus* needs to take these lipids from its host. Whether EgAgB apolipoproteins are directly involved in the uptake of fatty acids and sterols from host tissues remains to be elucidated, but most likely they contribute to transport these lipids within metacestode tissues. Delivery of sterols to metacestode target cells may be crucial for biosynthetic purposes (i.e. cholesterol for biological membranes), but also for triggering signaling pathways associated with parasite development and growth. In fact, signaling pathways involving sterol-responsive nuclear receptors are conserved from simple invertebrates to mammals and regulate metabolism and development [53]. For signalling actions host sterols may be modified by the parasite; indeed, a couple of putative steroid modifying enzymes have been found in the *E. granulosus* trasncriptome (our unpublished observations). Interestingly, about twenty nuclear receptors have been recently identified in *E. multilocularis* and *E. granulosus* and one of them displayed structural similarities to the DAF-12 subfamily, which binds cholesterol modified compounds and regulates cholesterol homeostasis and longevity in metazoans [54]. In addition, TAG are also a major component of EgAgB. TAG are not synthesised *de novo* by *E. granulosus*, but may be synthesised from building blocks obtained from the host as it occurs in other cestodes [33]. Metabolic studies have not been performed in *Echinococcus*, but evidence of TAG synthesis via α-glycerophosphate–phosphatidic acid-diglyceride pathway exists for the cestode *Hymenolepis diminuta* [55]. TAG are the major reserve of energy in animals, but no evidence on the operation of fatty acid oxidation pathways has been obtained in flatworms [33] including *Echinococcus* (our unpublished observations from the *E. granulosus* transcriptome). This scenario suggests that TAG mainly provide a source of fatty acids and glycerol for synthetic purposes via an enzymatic hydrolysis. Consistent with this view is the identification of two ESTs clusters (EGC01304 and EGC 03444) that encode putative lipases in the *E. granulosus* transcriptome data base. With respect to the high content of phospholipids in EgAgB (mainly phosphatidylcholine), these molecules likely play a structural role in the lipoprotein by exposing a polar outer surface required for lipoprotein solubilization in the aqueous milieu. Phospholipids are not synthesised *de novo* by flatworms, but they can be synthesised from building blocks obtained from the host (fatty acids and the head group) [33].

The uptake and delivery of lipids by lipoproteins require the existence of lipoprotein receptors in target cells. The existence of parasite EgAgB receptors as well as the use of host receptors by EgAgB would be needed for EgAgB-mediated lipid traffic. In this sense it is worth to mention that in *E. multilocularis* and *E. granulosus* genomes, antigen B gene cluster is flanked by *EmLDLR* or *EgLDLR* genes, which encode proteins that display significant sequence similarities to low density lipoprotein (LDL) receptors from other species, and contain one single class A LDL receptor domain [19]. The N-terminal end of the LDL receptor contains seven successive class A domains (a cysteine-rich repeat of about 40 amino acids), which are involved in the binding of LDL as well as very low density lipoprotein (VLDL) [56]. Furthermore, domains with homology to class A LDL receptor occur in related lipoprotein receptors such as VLDL receptor as well as LDL receptor-related protein/alpha 2-macroglobulin receptor [57], [58].

Overall, a new picture for EgAgB structure and function has emerged from this work. EgAgB is a complex 229 kDa lipoprotein capable of transporting a variety of lipid classes including essential lipids that are not synthesised by the parasite, such as fatty acids and sterols. EgAgB uptake and delivery of these lipids may not only contribute to biosynthetic purposes, but also to signalling events associated with parasite metabolism and development. Whether the hydrophobic ligand binding properties of EgAgB, reflected by its lipid class composition, are an intrinsic feature of cestode HLBP family remains to be determined.
